# Personalized prediction of incident hospitalization for cardiovascular disease in patients with hypertension using machine learning

**DOI:** 10.1186/s12874-022-01814-3

**Published:** 2022-12-17

**Authors:** Yuanchao Feng, Alexander A. Leung, Xuewen Lu, Zhiying Liang, Hude Quan, Robin L. Walker

**Affiliations:** 1 Centre for Health informatics, Department of Community Health Sciences, Cumming School of Medicine, University of Calgary, Calgary, AB Canada; 2 Libin Cardiovascular Institute, University of Calgary, Calgary, AB Canada; 3 Department of Medicine, Cumming School of Medicine, University of Calgary, Calgary, AB Canada; 4 Department of Mathematics and Statistics, University of Calgary, Calgary, AB Canada; 5 O’Brien Institute for Public Health and Alberta Health Services, 3280 Hospital Drive NW, Calgary, AB T2N 4Z6 Canada

**Keywords:** Administrative health data, Machine learning, Personalized prediction, Hypertension patients, Cardiovascular disease

## Abstract

**Background:**

Prognostic information for patients with hypertension is largely based on population averages. The purpose of this study was to compare the performance of four machine learning approaches for personalized prediction of incident hospitalization for cardiovascular disease among newly diagnosed hypertensive patients.

**Methods:**

Using province-wide linked administrative health data in Alberta, we analyzed a cohort of 259,873 newly-diagnosed hypertensive patients from 2009 to 2015 who collectively had 11,863 incident hospitalizations for heart failure, myocardial infarction, and stroke. Linear multi-task logistic regression, neural multi-task logistic regression, random survival forest and Cox proportional hazard models were used to determine the number of event-free survivors at each time-point and to construct individual event-free survival probability curves. The predictive performance was evaluated by root mean squared error, mean absolute error, concordance index, and the Brier score.

**Results:**

The random survival forest model has the lowest root mean squared error value at 33.94 and lowest mean absolute error value at 28.37. Machine learning methods provide similar discrimination and calibration in the personalized survival prediction of hospitalizations for cardiovascular events in patients with hypertension. Neural multi-task logistic regression model has the highest concordance index at 0.8149 and lowest Brier score at 0.0242 for the personalized survival prediction.

**Conclusions:**

This is the first personalized survival prediction for cardiovascular diseases among hypertensive patients using administrative data. The four models tested in this analysis exhibited a similar discrimination and calibration ability in predicting personalized survival prediction of hypertension patients.

## Background

Hypertension is the leading risk factor for preventable cardiovascular morbidity and premature death from cardiovascular disease (CVD) [1]. Accurate prediction of CVD can help to [2, 3], identify high-risk patients and therefore support clinical decision-making. Prognostic prediction has traditionally been based on the average event-free survival time in a population which is then projected onto an individual [4]. There is little research focusing on individual-level prediction.

In this study, we consider several important machine learning (ML) methods that produce the entire survival probability curve for individual patients. Recently, research has reported the risk analysis and survival prediction for cancer patients by machine learning techniques as well as on different input features and data samples. Weng el at. report using machine learning to improve accuracy of cardiovascular risk [5]. Results found that machine learning method improves accuracy of cardiovascular risk prediction, increasing the number of patients identified who could benefit from preventive treatment, while avoiding unnecessary treatment. Bharath et al. also found similar results in that machine learning improve prediction accuracy in CVD prediction model in an initially asymptomatic population [6]. Although machine learning methods have shown encouraging success on predicting some medical conditions, it has not been applied to individually CVD survival prediction in patients with hypertension by using routinely collected large digital electronic administrative health data. If the large administrative data set can be exploited using machine learning algorithm, it may open the way to optimise the use of collected administrative data to assist in predicting patents’ outcome, planning individualised patient care, monitoring resource utilization and improving institutional performance. Including comorbidity status, demographic data, lab test results and medication would improve assessment of prognosis and guide treatment decisions for hypertension patients.

Use of machine learning methods in clinical oncology has shown success [7, 8], but this methodology has not been more broadly applied to other clinical areas. Addressing this, the purpose of this study was to compare evaluate four ML approaches for personalized prediction of incident hospitalization for heart failure (HF), myocardial infarction (MI), and stroke among newly diagnosed hypertensive patients using routinely collected administrative health data. To our knowledge, this the first study to develop and validate different state-of-the-art ML models for individual CVD outcome prediction in hypertensive patients.

## Methods

### Data sources and study population

**Fig. 1:**
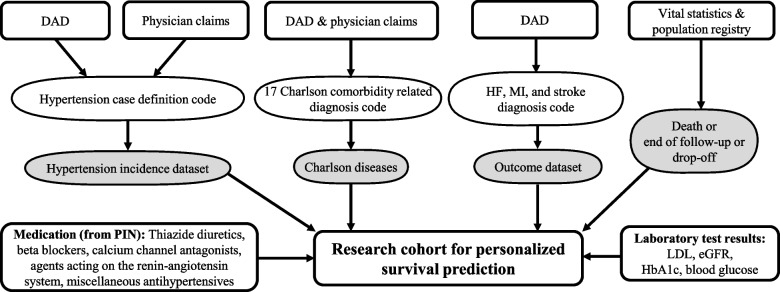
Linkage of six administrative health dataset to determine the study cohort. Note: DAD = Discharge Abstract Database, eGFR = Estimated glomerular filtration rate, HbA1C = Glycated haemoglobin (A1c), HF = heart failure, LDL = Low-density lipoproteins, MI = myocardial infarction, PIN = Pharmaceutical Information Network

A retrospective cohort was assembled using linked administrative health databases from Alberta Health with information including demographic and vital statistics, physician billing claims, medication dispensations, hospital separation data, and laboratory services (Fig. 1). These data have been used in previous studies and shown to be high-quality and comprehensive [9, 10].

**Fig. 2:**
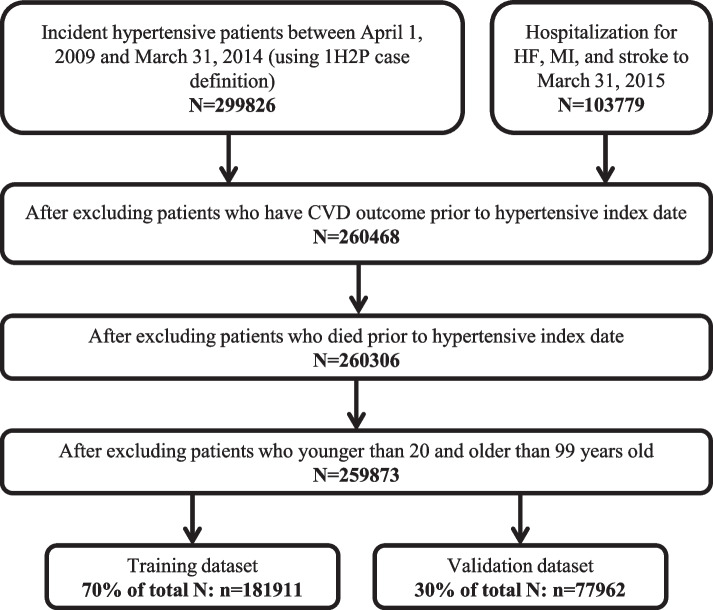
The flow for hypertension cohort identification. Note: 1H2P = 1 hospitalization or 2 physician claims within 2 years, CVD = Cardiovascular disease, HF = heart failure, MI = myocardial infarction

The study population included all newly diagnosed cases of hypertension aged 18 to 99 years who were residents of Alberta. We identified hypertension cases using a validated case definition of two physician claims within two years or one hospitalization with hypertension related diagnosis codes (ICD-9-CM: 401.x, 402.x, 403.x, 404.x or 405.x; ICD-10-CA: I10.x, I11.x, I12.x, I13.x or I15.x) [11]. The first date of the hypertension diagnosis (index date) was assigned to patients for case definitions with more than one hypertension diagnosis. We included patients who were identified to have hypertension between April 1, 2009, to March 31, 2014 (excluding those who had any code for hypertension between April 1, 2006, to March 31, 2009, thus allowing for a 3-year washout period, and thereby limiting the cohort to only newly diagnosed cases). Patients with prior CVD were also excluded. Cohort assembly is summarized in Fig. 2.

### Outcome

The outcome of interest was incident hospitalization for any CVD, including HF, MI or stroke, identified using validated case definitions: HF (ICD-10-CA: I09.9, I11.0, I13.0, I13.2, I25.5, I42.0, I42.5-I42.9, I43.x, I50.x, P29.0), MI (ICD-10-CA: I21.x, I22.x, I25.2), and stroke (ICD-10-CA: H34.1, I60.x, I61.x, I63.x, I64.x, G45.x) [12]. The time of event was defined as the first occurrence of hospitalization for HF, MI or stroke. We followed patients from initial hypertension diagnosis until the time of the outcome event, death, emigration out of the province, or the end of the study, up to March 31, 2015. If a patient experienced more than one event only the first event was counted as the incident event. Outcome event rates were calculated per 1000 person-years based on a maximum 6-year follow-up period.

### Predictors

Potential predictors were selected a priori based on previous studies and clinical reasoning [13]. Age was categorized into four age groups as the predictor in this study. Patients’ demographic information, such as sex, region of residence was also used as predictors. The number of Charlson comorbidities present in each patient was categorized into “0, 1-2, or ≥ 3.” Fasting blood glucose, estimated glomerular filtration rate (eGFR), cholesterol levels and Glycated haemoglobin (A1c) (HbA1c) was determined between hypertension index date and outcome date [14, 15]. Test results outside the standardized reference intervals were used (Blood glucose > = 7.0 mmol/L, eGFR < 60 mL/min/1.73m^2^, Cholesterol levels > 3.5 mmol/L, HbA1c > =6.5%) [16].

The following categories of antihypertensive medications have been shown to reduce cardiovascular risk and were identified using the anatomical therapeutic chemical (ATC) classification system: beta blockers (ATC codes in category C07, excluding C07AA07, C07AA12 and C07AG02); agents acting on the renin-angiotensin system (ATC codes in category C09); thiazide diuretics (ATC codes in category C03, excluding C03BA08 and C03CA01); calcium channel antagonists (ATC codes in category C08); and miscellaneous antihypertensives (ATC codes in category C02, excluding C02KX01) [17]. Respondents were categorized as using antihypertensive medication if an ATC code corresponded to the above list between the hypertension index date and outcome index date.

### Statistical analysis

The study cohort was randomly divided into training (70% of total: *n* = 181,911) and validation (30% of total: *n* = 77,962) sets (Fig. 2). Multicollinearity between predictor variables was assessed using condition indices and variance proportions. Those with significant correlation were removed from the model. The linear multi-task logistic regression (LMTLR) model is an alternative to the Cox’s proportional hazard (CoxPH) model. It can be seen as a series of logistic regression models built on different time intervals to estimate the probability that the event of interest happened within each interval. The constructed LMTLR included 25 features and 50 intervals in this study. The neural multi-task logistic regression (NMTLR) allows the use of Neural Networks within the original multi-task logistic regression (MTLR) design. We used the same 25 features, 100 neurons in the first hidden layer and 100 neurons in the second hidden layer, and one output neuron before input to LMTLR. The random survival forest (RSF) is an extension of the Random Forest model that can take into account censoring individuals. We used 50 trees, the maximum depth of 5 and minimum number of samples required to be at a leaf node at 20 for the model development. The CoxPH is a semi-parametric model that focuses on modeling the hazard function, by assuming that its time component and feature component are proportional over time. The maximum number of iterations in the Newton optimization in this model was 600.

#### Model validation

The final survival prediction model was tested within the test dataset for those four models (LMTLR, NMTLR, RSF, CoxPH) [18]. The actual and predicted number of patients that experienced the CVD event at each time t was compared by computing the actual survival function of the validation data, which can be obtained using the Kaplan-Meier estimator and compare it to the average of all predicted survival functions [18]. Root mean squared error (RMSE) and mean absolute error (MAE) was used to provide the comparison as well as the performance metrics between the actual and predicted number of hypertensive patients experiencing a CVD event at each time, t. Model accuracy was assessed using discrimination (concordance index (C-index)) and calibration (Brier score).

Analyses were conducted using SAS version 9.4 [19], R software version 3.5.1 [20] and Python version 3.7.6 [21]. Descriptive statistics were generated by SAS (Tables 1 and 2). The package ‘survival’ in R was used to produce Fig. 1 for survival analysis. ‘PySurvival’ in Python was used for ML model analyses. All the methods were performed in accordance with relevant guidelines and regulations.

**Table 1:** Population characteristics

Characteristics	No. (%), Mean or Median (IQR)	
	*N* = 259,873 (899,392.8 person-years)	Percentage (95% CI)
Age in years (Mean ± SD)	56.6 ± 14.0	
Median (Q1, Q3)	56.1 (47.2, 65.8)	
Age groups (years)		
20–49	84,032	32.3 (32.2–32.5)
50–64	106,526	41.0 (40.8–41.2)
65–74	42,107	16.2 (16.1–16.4)
75–99	27,208	10.5 (10.4–10.6)
Sex		
Female	122,233	47.0 (46.8–47.2)
Male	137,640	53.0 (52.8–53.2)
Region of residence		
Rural	42,669	16.4 (16.3–16.6)
Urban	217,204	83.6 (83.4–83.7)
Number of Charlson Comorbidities		
0	175,227	67.4 (67.3–67.6)
1–2	69,443	26.7 (26.6–26.9)
≥ 3	15,203	5.9 (5.8–5.9)
Charlson comorbidities		
Peripheral vascular disease	4721	1.8 (1.8–1.9)
Dementia	5008	1.9 (1.9–2.0)
Chronic pulmonary disease	38,808	14.9 (14.8–15.1)
Rheumatologic disease	4849	1.9 (1.8–1.9)
Peptic ulcer disease	4406	1.7 (1.7–1.8)
Mild liver disease	5010	1.9 (1.9–2.0)
Diabetes without chronic complications	20,065	7.7 (7.6–7.8)
Diabetes with chronic complications	5247	2.0 (2.0–2.1)
Hemiplegia or paraplegia	525	0.2 (0.2–0.2)
Renal disease	5344	2.1 (2.0–2.1)
Any malignancy, including leukemia and lymphoma	17,208	6.6 (6.5–6.7)
Moderate or severe liver disease	583	0.2 (0.2–0.2)
Metastatic solid tumor	2294	0.9 (0.9–0.9)
Lab test results		
LDL-cholesterol (> 3.5 mmol/L)	87,633	33.7 (33.5–33.9)
Blood glucose (≥7.0 mmol/L)	53,337	20.5 (20.4–20.7)
eGFR (< 60 mL/min/1.73m^2^)	63,697	24.5 (24.4–24.7)
HbA1c (≥6.5%)	36,016	13.9 (13.7–14.0)
At least one lab test	157,934	60.8 (60.6–61.0)
Medications		
Thiazide diuretics	64,248	24.7 (24.6–24.9)
Beta blockers	47,089	18.1 (18.0–18.3)
Calcium channel antagonists	62,112	23.9 (23.7–24.1)
Agents acting on the renin-angiotensin system	174,891	67.3 (67.1–67.5)
Miscellaneous antihypertensives	5245	2.0 (2.0–2.1)
≥ 1 of the medications listed above	209,729	80.7 (80.6–80.9)
Follow-up years (median (Q1, Q3))	3.6 (2.2, 4.8)	

Note: *CI* confidence interval, *IRQ* interquartile range, *Q1* first quartiles, *Q3* third quartiles, *SD* standard deviation, Number of Charlson comorbidities: excluding CVD related comorbidities and HIV/AIDS

**Table 2:**
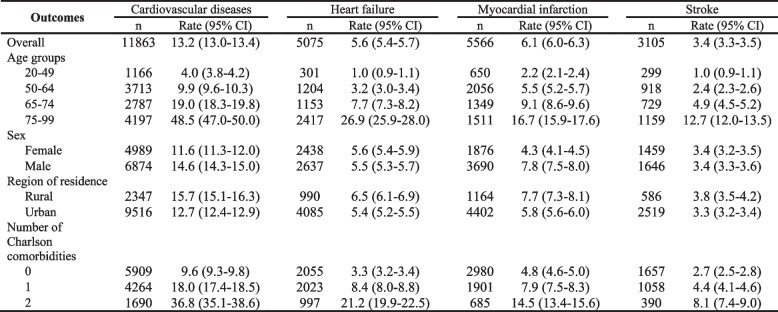
Hospitalization for incident cardiovascular disease (95% CI) among Albertan adults with newly diagnosed hypertension

## Results

### Cohort characteristics

We identified 299,826 newly diagnosed hypertensive patients between April 1, 2009, and March 31, 2015. After applying exclusion criteria, there was a total of 259,873 patients with 899,393 person-years of follow-up and collectively with 11,863 events over a median follow-up time of 3.5 years (inter-quartile range 2.2 to 4.8 years). The incidence rate was 13.4 CVD hospitalizations per 1000 person-years. Among the study population 9182 (3.5%) patients died within the study period. The mortality rate during the follow-up period was 10.0 per 1000 person-years (95% CI: 9.8 to 10.2 per 1000 person-years).

The median age of newly diagnosed hypertension patients was 56.1 years (26.7% were older than 65 years).and 83.6% resided in urban areas. The majority of patients had isolated hypertension without other major comorbidities, but up to one-third had at least one non-cardiovascular Charlson comorbidity, with diabetes being the most common, being present in around 1 in 10 people (9.7%). Nearly two-thirds of patients had at least one laboratory test of interest completed. An elevated LDL-cholesterol (33.7, 95% CI:33.5–33.9), elevated fasting blood glucose (20.5, 95% CI:20.4–20.7), and presence of renal dysfunction (24.5, 95%CI: 24.4–24.7) were the most common laboratory abnormalities. Most patients were dispensed with at least one antihypertensive medication (80.7, 95% CI: 80.6–80.9). Of these, the majority received an angiotensin converting enzyme inhibitor or angiotensin II receptor blocker (67.3, 95% CI: 67.1–67.5), followed by thiazide diuretics (24.7, 95% CI: 24.6–24.9), calcium channel blockers (23.9, 95%CI:23.7–24.1), and beta-blockers (18.1, 95%CI:18.0–18.3) (Table 1, locate at the end of the document text file).

The crude incidence of composite CVD hospitalization was 13.2 (95%CI: 13.0–13.4) per 1000 person-years. Hospitalization for MI was most common (6.1 (95%CI: 6.0–6.3) per 1000 events per person-years), followed by HF (5.6 (95%CI: 5.4–5.7) events per 1000 person-years), and lastly stroke (3.4 (95%CI: 3.3–3.5) events per 1000 person-years) (Table 2, locate at the end of the document text file). The composite CVD hospitalization rate was higher for men, and this was driven by an excess risk of MI. Hospitalizations were most common in patients above the age of 75 years, those residing in rural locations, and individuals with at least two other Charlson comorbidities both for the composite outcome and its individual components.

**Fig. 3:**
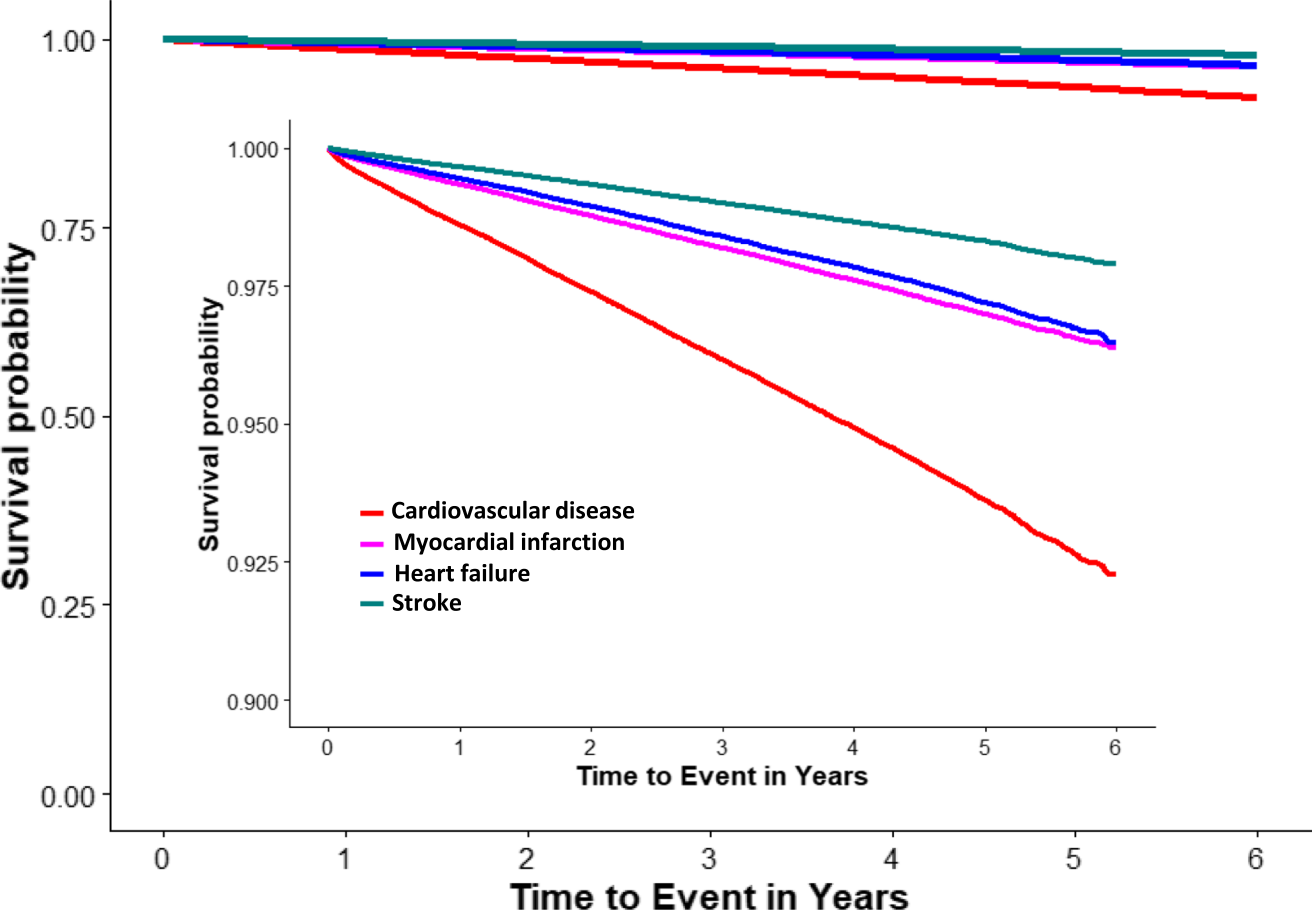
Kaplan-Meier estimate for incident hypertensive patients stratified by cardiovascular disease (any one of heart failure, myocardial infarction and stroke), MI, HF and stroke. Note: HF = heart failure, MI = myocardial infarction

Figure 3 shows Kaplan-Meier plots of the cumulative probability of being free of hospitalization for any CVD, HF, MI, and stroke as a function of survival time among newly diagnosed hypertension patients. MI had the lowest cumulative probability in the entire survival period when compared with HF and stroke.

### Model performance

Table 3 shows a comparison of the actual and predicted number of hypertension patients experiencing CVD events at each time point for all four models. The RSF model had the lowest RMSE at 33.94 and the lowest MAE at 28.37, indicating the best fit. By plotting the individual’s survival function over time, we compared the survival probability of developing CVD events among individual hypertensive patients. Table 4 shows the results when applying all four models in our hypertension cohort. Overall, all the models had a C-index > 0.70 and Brier score < 0.25 [22], representing a strong predictive ability in the validation set. Adding two layers of neural network before LMTLR, the NMTLR model had the highest C-index (0.82) and lowest Brier score (0.02); the best discrimination and calibration of all the models for event-free survival prediction. This suggests the NMTLR is most accurate and outperformed the other models in predicting CVD outcomes for incident hypertensive patients.

**Table 3:** Comparison of predicting the number of incident hypertensive patients diagnosed with cardiovascular disease(s) using predicted survival functions in multiple models (LMTLR, NMTLR, RSF and CoxPH)

Model performance	Models			
	LMTLR	NMTLR	RSF	CoxPH
RMSE	508.92	143.49	**33.94**	58.55
MAE	383.63	132.54	**28.37**	49.80

Note: *CoxPH* Cox’s proportional hazard, *LMTL* linear multi-task logistic regression, *NMTLR* neural multi-task logistic regression, *RSF* random survival forest, *RMSE* root mean squared error, *MAE* mean absolute error

**Table 4:** Summary of the results that measured by C-index and Brier score

Model performance	Models			
	LMTLR	NMTLR	RSF	Cox PH
C-index	0.7792	**0.8202**	0.8146	0.8165
Brier score	0.0350	**0.0243**	0.0343	0.0340

Note: *CoxPH* Cox’s proportional hazard, *LMTL* linear multi-task logistic regression, *NMTLR* neural multi-task logistic regression, *RSF* random survival forest, *C-index* concordance index

### Personalized prediction

**Fig. 4:**
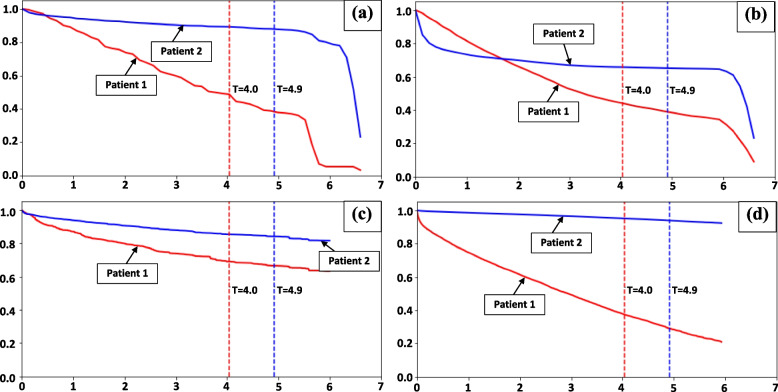
Personalized survival prediction by using (**a**) LMTLR model, (**b**) NMTLR model, (**c**) RSF model and (**d**) CoxPH model when randomly identifying two patients as an example. X axis is survival time in years, Y axis is survival probability. Patient one (red line) is a short survivor who lives for 4.0 years from diagnosis of hypertension to CVD, while patient two (blue line) is a long survivor whose survival time is censored at 4.9 years. Patient one developed CVD after 4.0 years diagnosed as hypertension, however, patient two was censored at 4.9 years after diagnosed as hypertension patient. The bottom position of survival time text (4.0 and 4.9) on the pictures correspond to 50% survival probability horizontal location line. Note: CoxPH = Cox’s proportional hazard, LMTL = linear multi-task logistic regression, NMTLR = neural multi-task logistic regression, RSF = random survival forest

Figure 4 visualizes the LMTLR, NMTLR, RSF and CoxPH models for two representative patients from the validation set. Patient one (red line) had a short event-free survival of 4.0 years from diagnosis of hypertension until being hospitalized for CVD, while patient two (blue line) had a comparatively longer event-free survival of at least 4.9 years before being censored. Patient one developed CVD after 4.0 years diagnosed as hypertension, however, patient two may have been lost to follow-up or did not develop CVD at the end of the study or death until 4.6 years after diagnosed as a hypertensive patient. The median survival time (50% survival probability) as a point estimation for survival time predication was used in the study for personalized survival prediction performance evaluation. If the 50% survival probability is close to the survival time, the model has more accurate prediction performance. All four models performed well in predicting the prognosis for patient one whose 50% survival probability corresponded with the actual observed 4.0 years of event-free survival. However, only the NMTLR model provided accurate prediction of 50% survival probability for patient two who was lost survival information at 4.9 years in this study. For other models, take the LMTLR for example, in fig. 4(a), the survival probability for patient 2 at 4.9 years survival time is near 90%, and this patient’s 50% survival probability is nearby 6.6 years. Although patient 2 passed the 50% survival probability after the 4.9 years in image (a), this patient’s 50% survival probability does not close to the 4.9 survival years, which indicate the model could not well predict this patient’s survival information in this model. The NMTLR was able to handle the presence of censoring better than the other models. Moreover, the individual survival curves for these two patients intercrossed at the beginning of the observation period. This may reflect the real situation that patient two has worse health condition or perhaps the patient is treated and controlled one year after being diagnosed with hypertension. Patient two have a pretty flat curve in the following period, however patient one became worse in the whole follow up period. The CoxPH model was unable to properly handle censoring, as represented by a horizontal survival probability line for patient two.

**Fig. 5:**
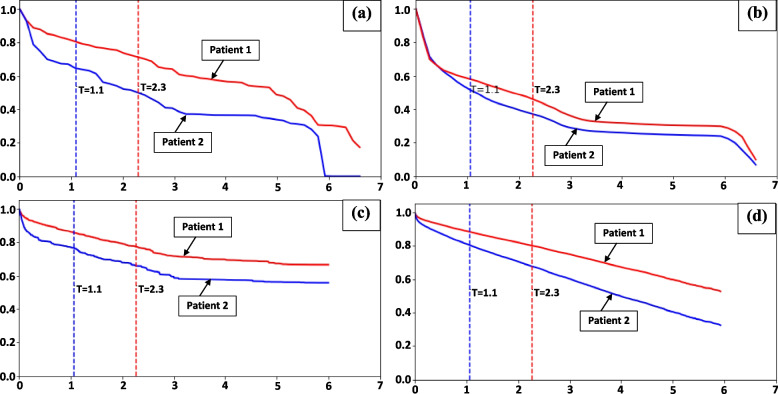
Personalized survival prediction by using (**a**) LMTLR model, (**b**) NMTLR model, (**c**) RSF model and (**d**) CoxPH model when randomly choosing two patients who have CVD outcome at 1.1 years and 2.3 years as an example. X axis is survival time in years, Y axis is survival probability. Hypertension patient one (red line) was diagnosed as CVD outcome at 2.3 survival years while hypertension patient two (blue line) was diagnosed as CVD outcome at 1.1 survival years. The bottom position of survival time text (1.1 and 2.3) on the pictures correspond to 50% survival probability horizontal location line. Note: CoxPH = Cox’s proportional hazard, LMTL = linear multi-task logistic regression, NMTLR = neural multi-task logistic regression, RSF = random survival forest

Figure 5 shows the prediction results for two patients who had CVD outcome at 1.1 years and 2.3 years, respectively. Patient one had a hospitalization for CVD at 2.3 survival years while patient two was had hospitalization for CVD at 1.1 survival years. All of those four models can discriminate the two patients’ survival time versus survival probability. Patient two’s survival curve was always lower than patient one and reached 50% survival probability faster than patient one. For NMTLR, the actual survival time corresponded closely to the estimated survival probability for both patients. For patients two, the survival curve was consistently lower than patient one, and the 50% survival probability occurred earlier for the first patient. Only NMTLR correctly predicted the survival time for patient one at 2.3 years and patient two at 1.1 years, based on the projected 50% survival probability. Moreover, NMTLR also had the smoothest survival curves with distinct shapes predicted for the two patients, while the CoxPH model predicted survival curves with similar shapes because of the proportional hazard assumption. For RSF model, we observed that the survival function was monotonically decreasing and parallel. This is likely due to both patients being in the same tree branch node in the model development process.

## Discussion

In this study, we explored the performance of ML models on predicting incident hospitalization for CVD distribution in patients newly diagnosed with hypertension using administrative data. All models were developed and validated using the same training dataset. All the four models had high discrimination with C-index > 0.70 and good calibration with Brier score < 0.25. We showed how ML models can be applied to accurately predict the occurrence of hospitalization for CVD on both population and individual levels.

For population use, the ML models can predict the number of patients with events at each time point using survival functions, similar to traditional regression methods. The RSF model had the best performance for population-based prediction compared to the other three models. Moreover, the RMSE and MAE were quite small in the RSF model, indicating that the prediction results were relatively constant during the 6-year follow-up period. As we did not force the use of any particular variables, the RSF algorithm was allowed to include any variables available in the administrative dataset that were associated with risk of hospitalization for CVD, which likely made the model more accurate than other models based on linear regression (LMTLR, NMTLR and CoxPH) for the population-based prediction [23, 24].

In terms of individual-level prediction, the NMTLR model had the highest C-index and lowest Brier score which means it had the best discrimination and calibration for individual survival prediction [25]. This may be because of the unique properties of using a neural network [26]. Neural networks require initialization and adjustment of many individual parameter to optimize the performance of the classification [27]. The NMTLR model that combines the neural network and multi-task logistic regression together was developed empirically and can be best fit for the training data in our study. NMTLR models the survival function by combining multiple local logistic regression modes in a dependent manner followed by a two layers neural networking procedure. This allows it to handle censored observations and the time-varying effects of features naturally to provide better results compared to the model which will only use fully observed (uncensored) instances (like CoxPH) [23]. The combination of neural network and multiple task logistic regression in NMTLR allows the model to build a nonlinear statistical data modeling tool to deal with complex relationships and has shown better predictive performance than the other three models [28].

## Strengths and limitations

To our knowledge, this is the first study to develop and validate models for individual CVD outcomes among patients with hypertension using administrative data. Utilizing administrative health data provides the opportunity to: 1) utilize risk factors that are routinely collected; and2) adopt the methods into existing hypertension and cardiovascular care practice and programs that are relevant for precision medicine. Further, there is considerable potential to use this data to improve clinical care cross a spectrum of chronic diseases. Our study results support that large administrative data provides sufficient statistical power to develop and validate predictive algorithms with a larger set of risk factors and greater specification of those risks, which in turn generate distinct survival probability for a wide range of health profiles or populations. Importantly, for individual-level prediction, our finding suggests that NMTLR has the best discrimination and calibration performance when compared to the other three models.

There are limitations to this study. Firstly, most patients in the study were followed up for 3 years, which may not be adequate to capture all CVD outcomes, especially for those younger and have a small number of comorbidities. Secondly, this study was retrospective and conducted in a single cohort. Further study is required to demonstrate generalizability of our findings. Thirdly, there are many important factors, such as blood pressure and other CVD medications, that were not included due to the limitations of administrative health data used in this study. Lastly, this study did not fully take into account missing data. Variables were included in the model even if one patient had a single value in the chart. This may have some-what diminished our predictive accuracy; however, a strength of this approach is that it represents the true nature of administrative health data with minimal transformations and with no data imputations. Another consideration is that we elected to define hypertension patients using a validated diagnosis codes with a high sensitivity and specificity. This methodology that using 1 hospitalization and 2 physician claims algorithm for hypertensive patients’ definition could represents a more easily deployable solution to cohort building and model development.

## Conclusions

This study demonstrated that four ML models utilizing administrative health data exhibited similar high discrimination and calibration in predicting incident hospitalization for CVD among hypertensive patients. Specifically, the NMTLR model had the best individual survival prediction and the RSF model had the best population survival prediction. Improved prediction of outcome has the potential to help clinicians make more meaningful decisions about treatment. Importantly, this study makes use of administrative health data that is already routinely collected but underexploited by clinical health systems. While ML methodologies have many advantages, to truly improve patient care and outcomes, methods for teasing out causal relationships will remain an important part of the health care and biomedical armamentarium.

## Data Availability

Data for this research are available from the corresponding author on reasonable request.

## Abbreviations

CVD: cardiovascular disease
ML: machine learning
HF: heart failure
MI: myocardial infarction
DAD: discharge abstract database
eGFR: estimated glomerular filtration rate
HbA1c: Glycated haemoglobin (A1c)
LDL: low-density lipoproteins
PIN: pharmaceutical information network
ATC: anatomical therapeutic chemical
1H2P: 1 hospitalization or 2 physician claims within 2 years
LMTLR: linear multi-task logistic regression
CoxPH: Cox’s proportional hazard
NMTLR: neural multi-task logistic regression
MTLR: multi-task logistic regression
RSF: random survival forest
RMSE: root mean squared error
MAE: mean absolute error
C-index: concordance index
CI: confidence interval
IRQ: interquartile range
Q1: first quartiles
Q3: third quartiles
SD: standard deviation

## References

[1] Frieden TR, Jaffe MG (2018). Saving 100 million lives by improving global treatment of hypertension and reducing cardiovascular disease risk factors. J Clin Hypertens.

[2] Krittanawong C, Zhang H, Wang Z, Aydar M, Kitai T (2017). Artificial intelligence in precision cardiovascular medicine. J Am Coll Cardiol.

[3] Leopold JA, Maron BA, Loscalzo J (2020). The application of big data to cardiovascular disease: paths to precision medicine. J Clin Invest.

[4] Sy JP, Taylor JM (2000). Estimation in a cox proportional hazards cure model. Biometrics..

[5] Weng SF, Reps J, Kai J, Garibaldi JM, Qureshi N (2017). Can machine-learning improve cardiovascular risk prediction using routine clinical data?. PLoS One.

[6] Ambale-Venkatesh B, Yang X, Wu CO, Liu K, Hundley WG, McClelland R (2017). Cardiovascular event prediction by machine learning: the multi-ethnic study of atherosclerosis. Circ Res.

[7] Krittanawong C, Virk HUH, Bangalore S, Wang Z, Johnson KW, Pinotti R (2020). Machine learning prediction in cardiovascular diseases: a meta-analysis. Sci Rep.

[8] McCradden MD, Baba A, Saha A, Ahmad S, Boparai K, Fadaiefard P (2020). Ethical concerns around use of artificial intelligence in health care research from the perspective of patients with meningioma, caregivers and health care providers: a qualitative study. CMAJ Open.

[9] Quan H, Chen G, Walker RL, Wielgosz A, Dai S, Tu K (2013). Incidence, cardiovascular complications and mortality of hypertension by sex and ethnicity. Heart..

[10] Metcalfe A, Neudam A, Forde S, Liu M, Drosler S, Quan H (2013). Case definitions for acute myocardial infarction in administrative databases and their impact on in-hospital mortality rates. Health Serv Res.

[11] Quan H, Khan N, Hemmelgarn BR, Tu K, Chen G, Campbell N (2009). Validation of a case definition to define hypertension using administrative data. Hypertension..

[12] Quan H, Chen G, Tu K, Bartlett G, Butt DA, Campbell NR (2013). Outcomes among 3.5 million newly diagnosed hypertensive Canadians. Can J Cardiol.

[13] Quan S, Chen G, Padwal RS, McAlister FA, Tran KC, Campbell NR (2020). Frequency of laboratory testing and associated abnormalities in patients with hypertension. J Clin Hypertens.

[14] Schwartz GL, Krakoff LR (2014). Diagnostic evaluation initial evaluation: laboratory testing. J Am Soc Hypertens.

[15] Rabi DM, McBrien KA, Sapir-Pichhadze R, Nakhla M, Ahmed SB, Dumanski SM (2020). Hypertension Canada’s 2020 comprehensive guidelines for the prevention, diagnosis, risk assessment, and treatment of hypertension in adults and children. Can J Cardiol.

[16] Pearson GJ, Thanassoulis G, Anderson TJ, Barry AR, Couture P, Dayan N (2021). 2021 Canadian cardiovascular society guidelines for the Management of Dyslipidemia for the prevention of cardiovascular disease in the adult. Can J Cardiol.

[17] Wilkins K, Gee M, Campbell N (2012). The difference in hypertension control between older men and women. Health Rep.

[18] Feng Y (2020). Personalized survival prediction of cardiovascular disease among hypertensive patients: a machine learning approach based on health administrative data [master thesis].

[19] Institute S (2016). The SAS system for Windows.

[20] Team RC (2013). R: a language and environment for statistical computing.

[21] Van Rossum G, Drake FL Jr. Python tutorial: centrum voor Wiskunde en Informatica. Amsterdam; 1995.

[22] Jachan M, Feldwisch Genannt Drentrup H, Posdziech F, Brandt A, Altenmüller D-M, Schulze-Bonhage A (2009). Probabilistic forecasts of epileptic seizures and evaluation by the Brier score. 4th European conference of the International Federation for Medical and Biological Engineering.

[23] Yu C-N, Greiner R, Lin H-C, Baracos V. Learning patient-specific cancer survival distributions as a sequence of dependent regressors. Adv Neural Inf Process Syst; 2011 24.

[24] Li Y, Wang J, Ye J, Reddy CK (2016). A Multi-Task Learning Formulation for Survival Analysis.

[25] Steyerberg EW, Vickers AJ, Cook NR, Gerds T, Gonen M, Obuchowski N (2010). Assessing the performance of prediction models: a framework for some traditional and novel measures. Epidemiology.

[26] Van der Heide E, Veerkamp R, Van Pelt M, Kamphuis C, Athanasiadis I, Ducro B (2019). Comparing regression, naive Bayes, and random forest methods in the prediction of individual survival to second lactation in Holstein cattle. J Dairy Sci.

[27] Ahmed FE (2005). Artificial neural networks for diagnosis and survival prediction in colon cancer. Mol Cancer.

[28] Zupan B, Demsar J, Kattan MW, Beck JR, Bratko I (2000). Machine learning for survival analysis: a case study on recurrence of prostate cancer. Artif Intell Med.

